# Improving Chirped Fiber Bragg Grating Resolution for Position-Sensitive Sensors in Shock- and Detonation-Driven Experiments

**DOI:** 10.3390/s26082566

**Published:** 2026-04-21

**Authors:** Tetiana Y. Bowley, Kimberly A. Schultz, Jonathan A. Hudston, Peter C. Klepzig, Christian R. Peterson, Joseph R. DeLoach, Todd O. Lundberg, Steve Gilbertson

**Affiliations:** Focused Experiments, Los Alamos National Laboratory, P940, Los Alamos, NM 87545, USA; tyb@lanl.gov (T.Y.B.); kims@lanl.gov (K.A.S.); hudston@lanl.gov (J.A.H.); pklepzig@lanl.gov (P.C.K.); chcpeter@lanl.gov (C.R.P.); jdeloach@lanl.gov (J.R.D.); tlundberg@lanl.gov (T.O.L.)

**Keywords:** chirped fiber Bragg gratings, crossed polarization, spatial resolution, temporal resolution, shock waves

## Abstract

Chirped fiber Bragg gratings (CFBGs) are robust diagnostic sensors that are widely used to track detonation-driven and shock wave propagation. CFBGs are inscribed with a linearly chirped periodic index of refraction changes that alter the Bragg wavelength along the length of the probe. The light return of each individual Bragg element is captured by a detector at a unique time to map the full reflected spectrum. The CFBG spectrum is measured with a dispersive Fourier transform of the reflected light that temporally stretches the spectrum to increase spatial resolution and make a one-to-one map of the wavelength on a time axis. Here, we propose an improvement of CFBG temporal resolution by incorporating two co-linear laser pulses with orthogonal polarization states and a 5 ns time offset. The two separate signals were split and tracked by two separate detectors. An oscilloscope captured good separation in the signals, and two separate spectrograms were generated and interleaved in the post-processing of the data. This novel technique doubled the CFBG temporal resolution and led to a doubled location resolution. As a proof-of-concept of this technique, the resolution improvement was compared between standard CFBG measurements and the two polarization states method on a position-sensitive CFBG sensor. CFBG resolution doubling will advance sensor capabilities and will have a direct impact on improving capture and analysis in dynamic, high-explosive experiments.

## 1. Introduction

Fiber Bragg grating (FBG) sensors are commonly used to track and measure shock waves and high-explosive detonation in experimental studies [1,2,3]. Over the years, FBG modifications were incorporated to improve grating strength, linearity, versatility, durability and sensitivity [4,5]. FBG sensors have been widely adopted in technology for many years, particularly in monitoring bridges [6], aerospace [7,8], and environmental testing [1,9]. Interest in FBG sensors has grown gradually in the scientific community since the 1970s [1]. This valuable diagnostic can measure changes in static and dynamic strain, displacement, pressure, and temperature in real time [1,4,10]. If an FBG is stretched or compressed, its grating periodicity changes accordingly (increases or decreases). The main advantages of FBGs include their light weight, compact size, quick response, and electromagnetic resilience [11]. The grating has a nonperiodic variation in the refractive index at a specific wavelength, the Bragg wavelength [12]. When a grating is illuminated by a light source, the FBG reflects a narrow light spectrum around the Bragg wavelength [12,13]. The reflected spectrum can then be recorded by a spectrometer.

A chirped fiber Bragg grating (CFBG) is a more complex FBG type in which the grating period changes along its length [14,15]. Grating period variations allow for modifications to the Bragg wavelength and refractive index along the grating length [11]. These unique properties make CFBGs a valuable diagnostic tool in fiber-optic communications, dispersion compensation, and broadband sensing [1,11,16,17]. The CFBG response relies not only on the strain or temperature applied to the whole length of the grating but also on the individual grating chirp [16]. The overall light return of CFBGs depends on the light return of each local value of the grating period [11,18]. Due to the varied length of the grating period, the reflected spectrum changes linearly with the grating length. CFBGs are more sensitive than standard FBGs and can detect spatial events with resolutions of less than 0.5 mm [11,19]. The limitations of using CFBG versus FBG sensors include more complex design and fabrication, low durability in harsh environments, and the higher costs of the interrogation system [1,20].

In shock physics experiments, the length of the typical CFBG is within the 10 mm to 200 mm range. CFBG sensors are commonly used in detonation velocity tracking [18], shock wave measurements, healthcare, and mechanical engineering [11]. Importantly, CFBG sensors can be applied to measurements of detonation wavefront positions in high explosives (HEs) to measure detonation velocity [21]. Detonation and shock wave experiments are fast, sub-microsecond, and often result in CFBG consumption [4,21]. The CFBG progressively gets shortened according to the velocity of detonation [11,21]. As the grating is destroyed, the stop band width is reduced until the whole CFBG is consumed.

Several approaches have been used to improve CFBG spatial and temporal resolution [3,21]. The spectrally integrated approach uses a linear CFBG that is deployed and tracks temporal changes in the CFBG total integrated signal [3,18,21]. The addition of a dispersive element temporally stretches the reflected spectrum and improves the spatial resolution of measurements along the grating’s length. This time-stretch approach creates a temporal streak in the return spectrum. It has been shown to be sensitive to immediate spectral changes in the reflected CFBG signal. This method shows a more accurate tracking of the grating stop band width and does not depend on spectral patterns in the amplified spontaneous emission (ASE) source [21]. Here, we describe a novel approach to increase the temporal and location resolution of position-sensitive CFBG sensors. We used the crossed polarization sensing of a CFBG to separate two signals and remove their temporal overlap by including a temporal offset of 5 ns and incorporating two orthogonal polarization states. This method could advance the sensing characteristics of CFBGs and enhance their temporal resolution and ability to measure event locations.

## 2. Results

Our experimental system was based on a previously developed CFBG diagnostic setup that tracks the velocity and temporal wavefront position of high-explosive (HE) detonations, in which a CFBG is consumed during the experiment [21,22]. The effective length of the grating shortens as the intense destructive shock travels along the HE. CFBG shortening results in a reduction in the stop band width, as measured on an oscilloscope. This system will produce results with measurements of the detonation position as a function of time. Sometimes the initial shock wave appears at a random place along the grating length and causes the CFBG to break prior to its consumption, and this complication can skew the results.

**Figure 1:**
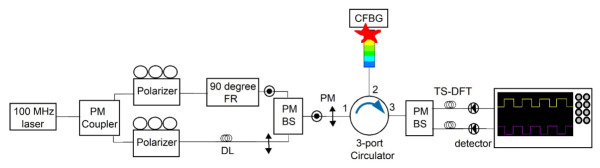
Schematic representation of cross-polarization CFBG. PM, polarization maintaining. BS, beam splitter. FR, Faraday rotator. DL, delay. TS-DFT, time-stretch dispersive Fourier transforms. MHz, megahertz. (1), (2), (3) Ports of the circulator.

We introduced several modifications to the traditional diagnostic setup to enhance the temporal resolution of CFBG sensors (O/E Land Inc., Lasalle, QC, Canada). We built a crossed polarization CFBG system using polarization-maintaining fibers (Figure 1). A 50:50 split of laser power (ELMO, MenloSystems, Martinsried, Germany) was achieved by sending a polarization-maintaining (PM) laser into a 50:50 beam splitter (OE Market, Galston, Australia). Each half of the light was directed into polarizers (Newport Optics, Irvine, CA, USA), with one branch going into a 90-degree Faraday rotator (OE Market, Galston, Australia) to obtain perpendicular polarization states. The signals from two orthogonal states were recombined using a circulator (OE Market, Galston, Australia) and were directed to the CFBG sensor. The two orthogonal sources were then sent to a polarized beam splitter to track two states with separate detectors (Optilab, LLC, Phoenix, AZ, USA). The 5 ns offset within the interferometer was achieved with a single 1 m PM jumper in the delay branch. This allowed for signal separation and increased the CFBG temporal resolution. To measure the offset in time between the two branches of the interferometer, a sub-100-femtosecond laser pulse was injected before the first coupler. The two outputs from the interferometer were measured on two separate 23 GHz bandwidth detectors and recorded on two channels of a 23 GHz DPO72304DX oscilloscope (Tektronix, Beaverton, OR, USA) recording at 50 GS/s. The initial laser pulse was found to have dispersed to 200 ps with a sub 100 ps risetime after the interferometer. The delay was measured to be 5 ns with an error of 40 ps. We used a trimmed 145 mm (150 mm pre-trimmed) chirped grating with a predefined chirp slope to monitor light return during the experiment. After the PM circulator, the two orthogonal states were separated by a second PM beam splitter. This was the end of the PM components. After the final PM beam splitter, the signals were sent through long delays (M2 Optics, Inc., Raleigh, NC, USA) to act as time-stretched dispersive Fourier transforms (TS-DFTs) of the pulses to get an accurate measurement of the spectrum shape. The length of the dispersion was chosen to ensure enough spectral broadening while also not causing pulse-to-pulse overlap between consecutive pulses from the mode-locked laser. After the PM interferometer, the dispersion modules were only there to act as a TS-DFT, and so maintaining the polarization states was unnecessary. Since the dispersion modules were not PM fiber, the polarization states were randomized after only a few meters of propagation. This averaged out the polarization mode dispersion in the fiber so that it did not need to be considered. Two separate detectors further transmitted two orthogonal signals to an oscilloscope. This setup helped to eliminate signal overlap and to increase temporal resolution. The detection of two distinct signals enabled us to perform independent analyses of two cross-polarized signals.

In general, CFBGs can be C-banded, L-banded, or in combination (C + L-banded). The centered wavelength for C-band CFBGs is 1550 nm, with a reflective range of 1533 nm–1568 nm. The L-band wavelength is 1605 nm. The stacking of C- and L-band gratings (1533 nm–1595 nm) on a single fiber is used to improve spatial resolution and length extension. For this experiment, we used C-band gratings with a 35 nm bandwidth and 150 mm length from OE Land, part number OEFBG-CHR-100. The reflected spectrum of a grating is checked prior to each experiment by means of a Luna Innovations optical backscatter reflectometer (OBR 4600; Luna Innovations, Roanoke, VA, USA) with a final resolution of 10 µm. This procedure is important for the calibration of the CFBG chirp slope and length. There can be a slight variation (1.5%) in OBR measurements between different gratings due to manufacturing variability. Therefore, OBR measurements of each sensor prior to each experiment ensure more precise data analysis afterwards.

**Figure 2:**
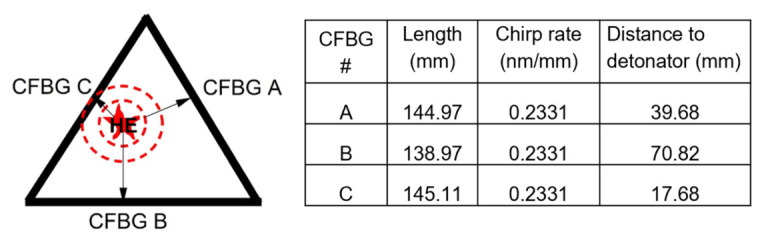
Schematic representation of position-sensitive sensor assembly. Detonator was positioned off-center to compare the detonation wave from three points. Table (right panel) lists CFBG sensors used in the experiment.

In this experiment, we positioned three CFBGs in a triangular configuration to record the detonation wave from a single, off-center detonator (Figure 2). This strategic positioning was aimed at monitoring outward-propagating shock waves generated by an HE detonator from different positions and distances. The total reflected spectrum was tracked along the CFBG length as it shortened due to shock processing. Each reflected signal had an apparent phase velocity dependent on the wave’s speed. The total spectrum was mapped to the wavelength and CFBG length, with the length of each grating measured prior to the beginning of the experiment. The difference in laser pulse time at the CFBG between two orthogonal signals was nominally 5 ns.

Overall, the reflected CFBG spectrum depends on the strain and temperature along the length of the sensor [11]. While the CFBG has an acrylate coating, stress and temperature influence can be neglected due to high pressure during detonation experiments. Additionally, shock pressure amplitude should be high enough to completely destroy the grating. If this is not the case, spectral shifts related to strain in the grating can be observed. In normal practice, gratings are positioned normal to the wavefronts since lateral strains introduce coupled behavior. In our position-sensitive sensor, the grating is parallel to the wavefronts. This truncates the grating at the point of impact and processes the remaining portion of the grating at an artificially high apparent velocity. Since the gratings are nearly parallel to the wavefront, coupled lateral strains would be difficult to interpret. It is expected that the position-sensitive sensor would work best when the strains are sufficiently high to process the grating; however, fielding in a lower-strain environment could be a subject for future work. An oscilloscope records the grating reflection intensity data as a voltage signal. A spectrogram is made by plotting each consecutive spectrum as a 2D array of spectrum versus time. The leading edge of the spectrogram is extracted and graphed as a function of position versus time [21].

**Figure 3:**
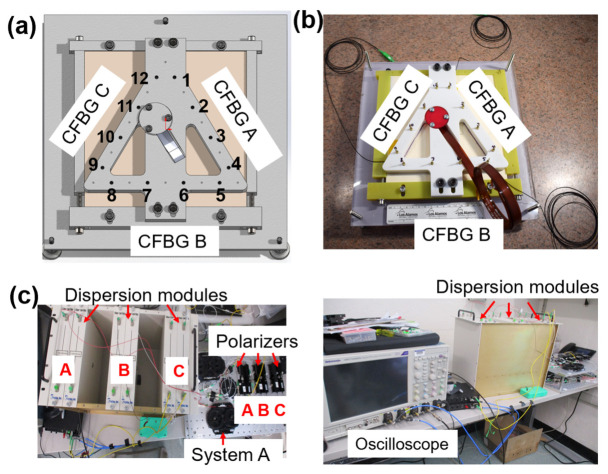
Experimental setup. (**a**) Schematic of the CFBG and pin positioning on the PBX-9501 slab shot; pin number and location are indicated; (**b**) picture of the final HE shot with probes just prior to execution; (**c**) pictures of the laser and recording setup indicating the location of different system components.

A plastic-bonded explosive (PBX-9501) slab was set up to measure an outward-propagating detonation front (Figure 3a,b). The experimental schematic (Figure 3a) shows two diagnostic systems in this experiment: piezo pins (type CA-1135; Dynasen Inc., Goleta, CA, USA) and CFBGs. These diagnostics recorded the detonation front as it traveled through the volume of the high-explosive charge. The total length of the PBX-9501 slab was 305 mm on each side, and it was 12.5 mm thick. The photographs of the assembled test show the details of the system components and the diagnostics location on the PBX-9501 surface (Figure 3b). Three systems were built on two breadboards: system A and all of the polarizers were set up on the top breadboard, while systems B and C were on the bottom one. The strategic placement of the polarizers on the top breadboard was intentional for easy access and final optimization of the polarization states prior to the shot.

A total of twelve piezo pins and three CFBGs were installed and fielded in this experiment. Piezo pins are commonly utilized in shock and detonation experiments for triggering and timing purposes [21]. With a sub-ns time response, they enable a high-speed voltage measurement after being destroyed by the massive shock pressure. We had a total of twelve pins, with four pins placed along each grating length (Figure 3a). The spacing between the pins was 45.3+/−0.5 mm (Figure 3a,b). CFBG information, such as chirp slope and sensor length, is presented in the table (Figure 2, right panel). A clock pulse from the CFBG laser was recorded for timing reference. Chromatic dispersion (Figure 3c) was achieved by introducing a Corning SMF-28e fiber spool of 6.5 km. As a rule, the single-mode optical fiber had an average chromatic dispersion fiber coefficient of 16.7 ps/nm/km at a wavelength of 1550 nm. A 6.5 km module provided pulse broadening for our gratings that measured ~7 ns on our oscilloscopes. With 6.5 km modules, we estimated a wavelength-to-time mapping coefficient of 108.6 ps/nm. With 35 nm bandwidth gratings, the dispersion module alone would have provided nearly 4 ns of delay, as measured on the oscilloscope. Combined with the dispersion of the grating itself, the 7 ns pulses that we recorded provided a good balance between spatial resolution and leaving a margin for additional spectral broadening due to unexpected strain.

A Luna OBR 4600 (Luna Innovations, Roanoke, VA, USA) was used to trim the CFBGs and measure the final length of the three gratings (Figure 2, right panel). The distance from the CFBGs to the detonator was purposefully different: 39.68 mm (system A), 70.82 mm (system B), and 17.68 mm (system C). CFBG C was the closest to the detonator. The CFBGs were mounted on the slab surface using M-Bond 200 quick-setting epoxy (DigiKey, Thief River Falls, MN, USA). FaroArm (InnovMetric Software Inc., Quebec City, QC, Canada) measurements provided the as-installed metrology of the CFBG, piezo pin, and detonator locator positions. Minor, nonlinear irregularities were expected to be present in the CFBG reflection due to the CFBG’s spectral non-flatness.

**Figure 4:**
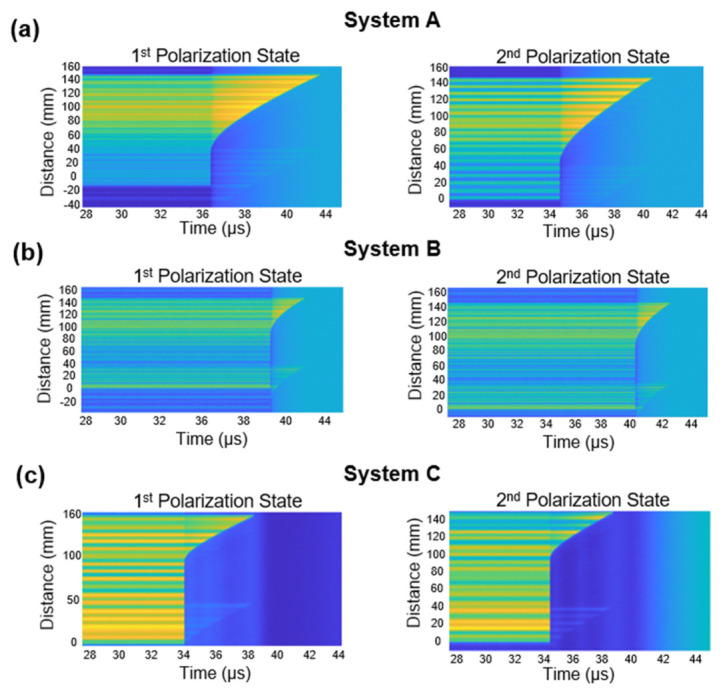
Spectrograms of the two polarization states. (**a**–**c**) Spectrograms were generated from the raw signals of the three CFBGs along the triangle. The time axis on the graphs is corrected to a common fiducial.

Each CFBG detected two independent signals from two polarization states. Two 2D spectrograms from two orthogonal signals for each CFBG were generated and are shown in Figure 4. A total of six spectrograms were generated (Figure 4a–c). Our results confirmed that all three CFBGs detected the presence of two polarization states in each experimental system. A slight crosstalk image is seen in each spectrogram due to the incomplete suppression of each polarization state. The remaining signal was small enough not to affect the ability to extract the leading edge in each measurement.

**Figure 5:**
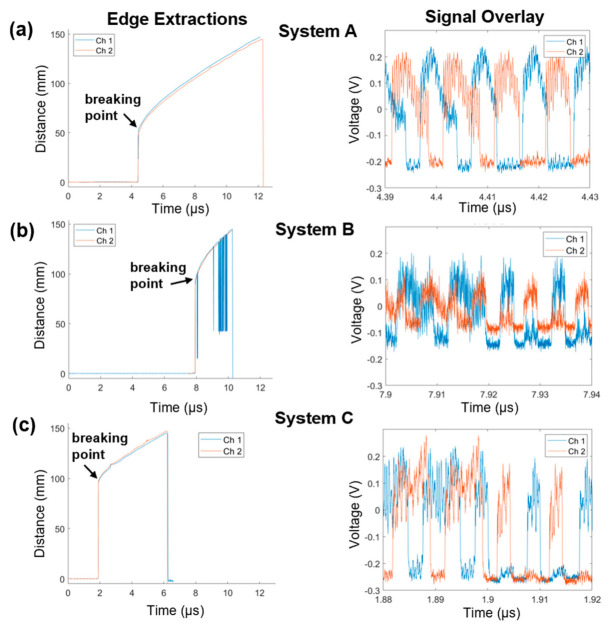
Edge extraction and signal overlay diagrams. Extracted leading edges of both polarization states (channel 1—blue, channel 2—red) are displayed in the left panel. The two extractions are shown as an overlay. Right panel contains an overlay of the raw detector signals (time corrected only) from each CFBG. (**a**–**c**) Time is corrected to a common timing fiducial for systems A, B, and C.

The detonation wave hit CFBG C first and CFBG B last. Extracted leading-edge diagrams (Figure 5a–c, left panels, time vs. detonation front position) were generated using the pulse-width detection technique and showed good agreement between both polarization states (overlay of the red and blue graphs). Signal overlap between the two signals was graphed for better visualization of the signal separation (Figure 5a–c, right panels). The velocities in Figure 5 are apparent velocities due to the wavefront curvature. When the curvature is small, the grating is processed more slowly than when the curvature is large. Additionally, the wavefront curvature is continuously increasing during this experiment. For these reasons, the velocity is not a meaningful representation of the HE velocity. While the wavefront curvature could be extracted from these results, it was beyond the scope of this proof-of-concept paper but could be the subject for future work. Timing corrections for each channel are listed in Table 1. This overlay method confirmed excellent agreement of data generated by probing a single CFBG with two non-simultaneous laser pulses at orthogonal polarization states. Overall, these results verify effective doubling of the CFBG temporal resolution.

**Figure 6:**
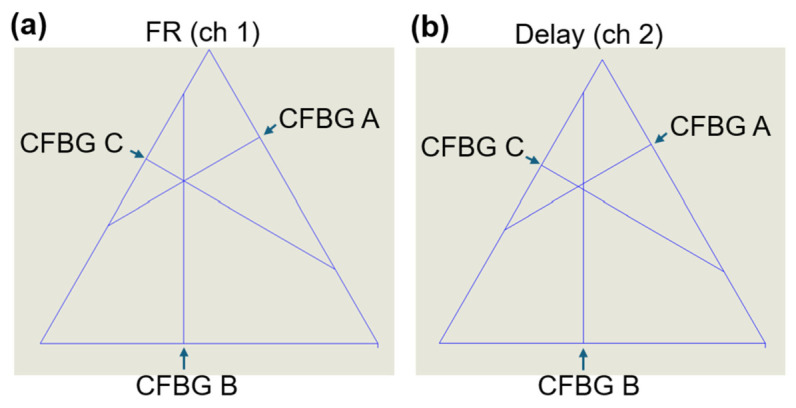
CFBG breaking point. The interception point was mapped using SolidWorks Premium v2024 for the FR (**a**) and delay (**b**) channels. Lines from the calculated breaking points were graphed at a 90° angle towards the triangle center. The exact breaking point of each grating is identified with an arrow. ch, channel. FR, Faraday rotator.

We determined the exact breaking point of each grating (Figure 6). The breaking point was calculated from the extracted edge diagrams as the first point from the vertical part of the graph to the gradual increase (Figure 5). From each breaking point, a line was drawn perpendicular to the grating. The point of intersection of the lines drawn from the breaking point showed the meeting location at the detonator position (Figure 6a,b). After the first beam splitter, the signal was equally divided between the Faraday rotator branch and the delay branch. Channel 1 for each system (system A, system B, and system C) recorded the FR branch of the system, while channel 2 measured the delayed signal branch. The second branch was intentionally delayed at the grating to capture the reflected spectrum nominally 5 ns after the first channel. The two orthogonal states were conserved due to the usage of PM fibers and are represented here as two separate triangles (Figure 6a,b). The calculated breaking point for each CFBG (distance and time) is presented in Table 2. The location resolution was created by plotting the lower and higher values from the breaking point (Table 3). The higher value was calculated as the next point on the graph after the breaking point and used to generate symmetric location resolution graphs (Table 3, third column).

**Figure 7:**
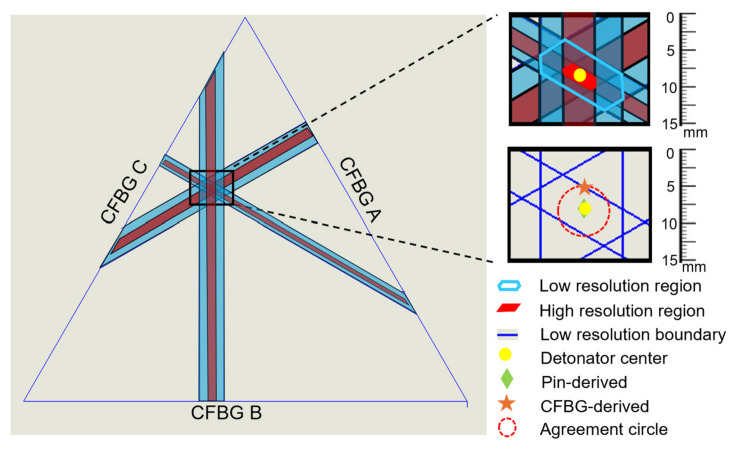
Measurement regions were generated by using the higher and lower values from the breaking point. Blue regions show low resolution, while red regions show high resolution.

Importantly, the presence of cross-polarization sensing of a CFBG doubled the temporal resolution, allowed for a doubling of the location resolution of the grating breaking point, and decreased the measurement uncertainty when the two channels were overlaid. Specifically, channel 2 measurement regions were plotted on top of channel 1’s breaking point to show resolution doubling (Figure 7, left panel). This is critical because improved CFBG temporal and location resolution provides more precise results and predictions for future experiments. With the two delayed polarization states, the effective repetition rate of the laser was increased from 100 MHz to 200 MHz. This allowed for sampling of the grating every 5 ns versus the 10 ns sampling expected with the lower repetition rate. Since the location resolution of an event occurring along the grating is tied to the sample rate, doubling the temporal resolution doubles the location resolution. In the insets of Figure 7, the upper figure shows a zoomed-in view of the high- and low-resolution regions of the three gratings. The small regions show the boundaries of the overlapping graphs and provide the best guess for the center of the initiation point. The overlapping region is the area of importance for determining the location of the detonation event. Using the standard temporal resolution, the region of overlap has an area that is seven times larger than the region of overlap measured with the cross-polarization CFBG technique. This reduces the uncertainty of the detonation point to an area much smaller than the detonator surface area. The yellow dot shows the as-measured center of the detonator. The high-resolution region pinpoints the initiation spot to +/−1.5 mm, while the low-resolution region determines the spot to within +/−3 mm. The lower inset figure in Figure 7 shows the same result but with the overlapping regions removed.

**Figure 8:**
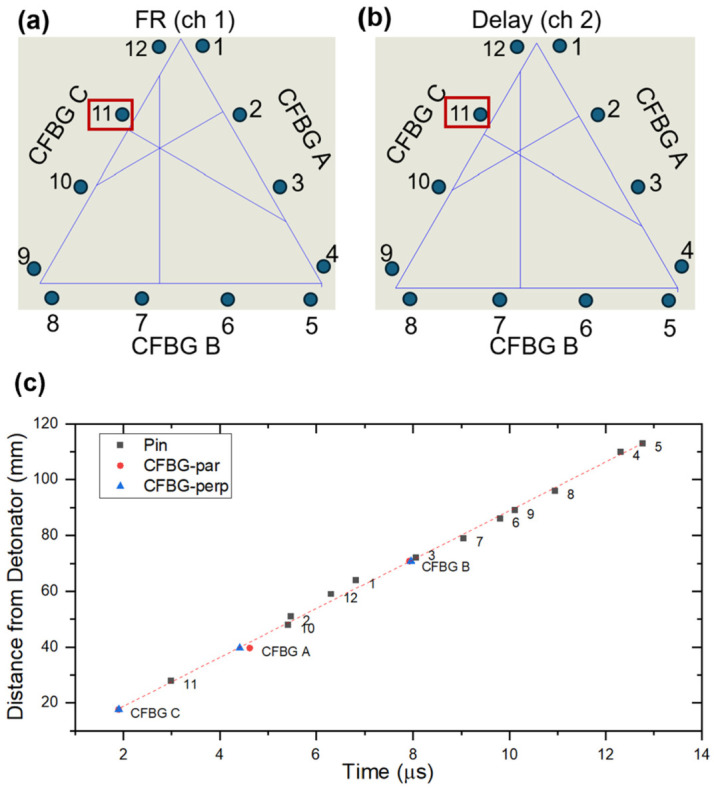
Piezo pin measurements. The location of twelve pins is shown for the FR (**a**) and the delay (**b**) channels. The closest pin to the detonator was #11 (red box). All fielded HE diagnostics were plotted as a function of distance to detonator versus time (**c**). ch, channel.

To confirm the CFBG results, we used piezo pins to measure the time of the shock front arrival in twelve locations around the outside of the CFBG triangle (Figure 3 and Figure 8a,b; Table 4). The closest pin to the detonator was pin #11 (Figure 8a, red box). Consistent with the shortest distance, the shock arrival time for this pin was the shortest (2.9879 us; Table 4). Using the technique of multilateration [23], the location of the detonator initiation was determined. For the measurement, the velocity of the detonation wave was determined using the timing offset and distance to the closest pin. The value was found to be 8.8 mm/µs. The lower inset in Figure 7 shows the location of the detonator initiation, as determined with all 12 pins. Using the same measured detonation velocity, the initiation point as calculated from the six measurements from the gratings was independently calculated with multilateration. The result is shown as the orange star in the inset of Figure 7. The combination of the calculated grating initiation point with the measured grating break locations gives an “agreement circle” that is centered around the measured pin results. The error between the diagnostics shows ~1.5 mm error. Gratings that are along the surface of detonating HE measure an apparent velocity which is the true velocity divided by the cosine of the angle between the detonation wavefront and the grating. For flat geometries, the angle is zero and the measured velocity is the true velocity. For curved geometries, the angle might not be zero or could change continuously, giving a much more complicated answer not easily accessible to multilateration. As the detonation velocity is not always known, particularly for curved geometries, multilateration may not be available for analysis in all experimental conditions. In those cases, the breaking points alone can be used, although with slightly higher uncertainties.

An overlay of all the HE detonation diagnostics, three CFBGs and twelve piezo pins was plotted as the distance to the detonator versus time (Figure 8c). Overall, there is consistency between the pin and the CFBG data.

## 3. Discussion and Conclusions

In this project, we demonstrated crossed polarization interrogation of a CFBG as a method to improve the temporal resolution in position-sensitive measurements. By using two temporally controlled and orthogonally separated polarization states, the effective sampling rate was increased two-fold (100 MHz to 200 MHz), resulting in an interleaved measurement of the same grating. The repetition rate of the laser and data collection was effectively doubled by staggering the pulses in time. The obtained perpendicular signals were analyzed, and separate spectrograms were generated (Figure 4). The extracted leading edge showed delayed sensing of the grating with the two polarization states, with a nominal 5 ns delay (Figure 5). We also calculated the exact point where each CFBG was broken and mapped it to the precise location of the detonator (Figure 6). Resolution doubling resulted in a reduction in the uncertainty regions between measurements (Figure 7) and improvement of the CFBG efficiency.

In general, the change between the fast and slow axes of the fiber is related to the beat length of the PM fiber type. The beat length of the fiber was 4–5 mm, which corresponds to a difference in the refractive index of 0.00034, which is less than 0.03% of the allowed index of refraction in SMF-28 and thus does not impact the results of this experiment. The modulation depth of the interference was reduced by using slice-by-slice spectral smoothing to improve the quality of the spectrograms. Combining the cross-polarization CFBG method with the position-sensitive sensor allowed us to measure the exact location of the detonation event. This would be useful for instances when the impact location is unknown, for example, the initiation of an HE by a metal fragment. Through the use of two polarization states, the region of uncertainty was found to be reduced by a factor of seven (high resolution area of 5.8 mm^2^ versus low resolution area of 46.2 mm^2^).

The improvements presented in this project can be understood in the context of existing approaches to enhancing CFBG resolution. Conventional methods for enhancing CFBG resolution predominantly rely on modifying dispersion or grating design [1,24,25]. While effective, these approaches are constrained by detector bandwidth and its signal-to-noise ratio or fabrication limitations of the grating. In contrast, the cross-polarization method increases the effective sampling rate by incorporating readily available polarization-maintaining components and a controlled delay line, thereby providing a practical temporal enhancement of existing CFBG-based diagnostic systems.

The optical response of the grating and the shock interaction geometry both influence the measured result. Furthermore, shock-loaded optical fiber experiences significant anisotropic transverse stress, resulting in transient birefringence and polarization-dependent spectral variations. However, the measurement presented in this project relies on grating truncation time rather than detailed spectral features. The strong agreement between orthogonal polarization channels demonstrates that the measurement is insensitive to birefringence-induced differences and confirms temporal localization of the truncation point under the conditions of this experiment.

The parallel orientation of the grating relative to the shock wave represents a departure from conventional CFBG fielding, where gratings are typically oriented normal to the shock wave for velocity measurements. In this configuration, position-sensitive localization is achieved through measurement of the grating truncation time, which corresponds directly to the interaction point. To validate this grating configuration, a slab-shot geometry was employed, producing a detonation front that is locally planar at the gratings, resulting in sufficiently sharp events that defined well-resolved truncation points. The consistency of the extracted leading edges across polarization channels supports the results. However, in more complex scenarios, such as strongly curved geometries or evolving shock wave fronts, the truncation event may become temporally convoluted, increasing uncertainty in its determination and complicating interpretation.

In comparison to well-established diagnostics such as piezo pins, the crossed-polarization CFBG approach provides an intrinsically safe, minimally intrusive method for distributed sensing along the length of the grating. While piezo pins offer high temporal precision at discrete locations, they may perturb the experiment and, therefore, may not be suitable for all configurations. Conversely, the cross-polarization CFBG method enables distributed measurements with reduced localization uncertainty and provides a practical means to enhance the temporal resolution of existing CFBG diagnostic systems.

While further work is needed to fully characterize cross-polarization CFBG performance in more complex configurations, these results establish a complementary technique for improving position-sensitive measurements in shock and detonation experiments, alongside existing diagnostic methods.

## Figures and Tables

**Table 1:** Calculated timing corrections for each channel. ch, channel. OBR, optical backscatter reflectometer.

Name	System Delay (μs)	OBR (ns)	Final Timing Correction (μs)
System A ch 1	31.906	109.4	32.015
System A ch 2	31.905	109.4	32.014
System B ch 1	31.912	108.07	32.027
System B ch 2	31.908	108.07	32.016
System C ch 1	31.905	109.42	32.014
System C ch 2	31.908	109.42	32.017

**Table 2:** CFBG exact breaking points and times. ch, channel.

Count	Distance from Fiducial (mm) for 145 mm CFBG	Breaking Point Time (μs)	Corrected Breaking Point Time (μs)
System A ch 1	44.2964	36.6391	4.6241
System A ch 2	44.368	36.430574	4.416574
System B ch 1	85.124	39.951954	7.924954
System B ch 2	82.593	39.972356	7.956356
System C ch 1	92.7092	33.914466	1.900466
System C ch 2	92.7092	33.921716	1.904716

**Table 3:** The exact breaking points and higher and lower values of each CFBG. ch, channel.

Count	Distance from Fiducial (mm) for 145 mm CFBG	Higher Value (mm)	Distance from the Break Point to the Next Value (mm)	Lower Value (mm)
System A ch 1	44.3	48.3	4	40.3
System A ch 2	44.37	45.97	1.6	42.77
System B ch 1	85.12	89.13	4	81.12
System B ch 2	82.59	83.59	1	81.59
System C ch 1	92.71	94.71	2	90.71
System C ch 2	92.71	93.5	0.79	91.92

**Table 4:** Pin data measurements: time of shock arrival, scope number, voltage, and attenuation.

Pin Number	Time (μs)	Scope Number	Voltage (V)	Attenuation (dB)
1	6.8289	1	0.016	26
2	5.4753	1	0.032	26
3	8.0616	1	0.032	26
4	12.3115	1	0.024	26
5	12.7716	2	0.056	26
6	9.8117	2	0.04	26
7	9.0495	2	0.04	26
8	10.9436	2	0.048	26
9	10.1095	3	0.032	26
10	5.4217	3	0.04	26
11	2.9879	3	0.056	30
12	6.3076	3	0.048	30

## Data Availability

The original contributions presented in this study are included in the article. Further inquiries can be directed to the corresponding author. Data is unavailable due to privacy.

## Abbreviations

The following abbreviations are used in this manuscript:

FBG	Fiber Bragg grating
CFBG	Chirped fiber Bragg grating
HE	High explosive
PBX	Plastic-bonded explosive
PM	Polarization maintaining
ASE	Amplified spontaneous emission
TS-DFT	Time-stretch dispersive Fourier transforms
